# Effects of the revised WIC food package on women’s and children’s health: a quasi-experimental study

**DOI:** 10.1186/s12884-022-05116-w

**Published:** 2022-11-02

**Authors:** Alice Guan, Akansha Batra, Rita Hamad

**Affiliations:** 1 Department of Epidemiology & Biostatistics, University of California San Francisco, 550 16th Street, 94158 San Francisco, CA USA; 2 Department of Family & Community Medicine, Philip R. Lee Institute for Health Policy Studies, University of California San Francisco, San Francisco, CA USA

**Keywords:** Child nutrition, Child public health, Food policy, Health policy, Maternal public health, Maternal nutrition

## Abstract

**Background:**

The Special Supplemental Nutrition Program for Women, Infants, and Children (WIC) was revised in 2009 to be more congruent with national dietary guidelines. There is limited research examining effects of the revision on women’s and children’s health. The objective of this study was to evaluate whether the revised WIC food package was associated with various indicators of physical and mental health for women and children.

**Methods:**

We used 1998–2017 waves of the National Health Interview Survey (N = 81,771 women and 27,780 children) to estimate effects of the revised WIC food package on indicators of health for both women (self-reported health and body mass index) and children (anemia, mental health, and parent-reported health). We used difference-in-differences analysis, a quasi-experimental technique that assessed pre-post differences in outcomes among WIC-recipients while “differencing out” the secular underlying trends among a control group of non-recipients.

**Results:**

For all outcomes evaluated for women and children, we were unable to rule out the null hypothesis that there was no effect of receiving the revised WIC food package. These findings were confirmed across several secondary analyses conducted to assess heterogeneity of effects and robustness of results.

**Conclusion:**

While we did not find effects of the revised WIC food package on downstream health indicators, studies using similarly robust methods in other datasets have found shorter-term effects on more proximal outcomes related to diet and nutrition. Effects of the modest WIC revisions may be less impactful on longer-term indicators of health, and future studies should examine the larger COVID-19-era expansion.

**Supplementary Information:**

The online version contains supplementary material available at 10.1186/s12884-022-05116-w.

## Introduction

The Special Supplemental Nutrition Program for Women, Infants, and Children (WIC) was established in the 1970s to promote the health of low-income pregnant and postpartum women and children up to age five by providing food, nutritional education, and referrals to medical and health services [1, 2]. It continues to be one of the largest social safety net programs in the United States, serving approximately 7 million participants each month [3]. In 2009, the WIC food packages for women and children were revised to better align with federal dietary guidelines [4, 5]. The revision expanded provisions for healthier food options, including a voucher for fruits and vegetables, and requirements for milk to be low-fat and grain products to be whole grain [1].

Since the 2009 WIC revision was implemented, several studies have documented the positive effects of the new food package on retail food availability [6–9], women’s and children’s dietary quality [9–13], and maternal nutrition [6]. There has been less research on downstream health effects, with studies in California and South Carolina finding improvements on perinatal and birth outcomes [14, 15] and studies in Tennessee finding improvements in children’s growth and cognitive development [16]. No studies to our knowledge have examined the downstream effects of the revision on women’s and children’s health at a national level.

The objective of this study was to address this gap by investigating the effects of the WIC food package revision on women’s and children’s health using a national sample and rigorous quasi-experimental methods. This study thereby illuminates the population-level impacts on the health of low-income families and has implications for the design and implementation of safety net policies and other interventions to address nutrition among vulnerable women and children.

## Methods

### Data and sample

The sample was drawn from the U.S. National Health Interview Survey (NHIS), an annual cross-sectional survey (see sample flowchart, Supplemental Fig. 1). We used the 1998–2017 waves of NHIS, since surveys prior to 1998 did not contain sufficient information on family relationships, and 2017 was the most recently available when analysis began. While demographic data are collected by the NHIS for all household members (N = 1,989,149 for 1998–2017), this study was restricted to women who reported having children under the age of one in the household to capture the postpartum period (N = 172,903) and to children under five (N = 70,047). Although pregnant women are also eligible for WIC, information on pregnancy was not regularly ascertained by NHIS throughout the study period, and our results are thus only generalizable to postpartum women. Additionally, women are only eligible for WIC during pregnancy and up to six months postpartum (for non-breastfeeding women) or one year postpartum (for breastfeeding women). Thus, restricting our sample to those with children under the age of one captures a likely eligible population. Next, we restricted the samples to those for whom data on WIC receipt was non-missing (N = 145,119 women and 61,133 children). We additionally restricted the samples to those whose household income was less than $75,000 to ensure that the control group of non-recipients was more comparable to the treatment group (N = 81,771 women and 30,798 children). For children, we observed that outcome trends were not parallel in earlier time periods, violating an assumption of difference-in-differences analysis (see additional details below). Thus, we additionally excluded observations which occurred more than 100 months prior to when the revision was implemented (N = 27,780). Not all outcomes were collected for all women or children, resulting in variation in the number of individuals included in each analysis (Supplemental Fig. 1).

### Exposure

The main exposure was whether women or children received the revised WIC food package. The implementation of the WIC revision was staggered across states throughout 2009, with some states implementing the changes as early as January 2009 and others as late as November 2009 [17]. The NHIS assessed whether an individual had received WIC benefits via self-report. Among WIC recipients, we considered women and children to have received the revised food package if they reported WIC receipt after the revision was implemented in their state.

### Outcomes

For women, we assessed two measures of physical health. First, we evaluated change in self-reported health over the prior year, which was assessed by the question, “Compared with 12 months ago, would you say your health is better, worse, or about the same?” We dichotomized this variable as better or about the same versus worse. This question has been previously included as a part of the Short-Form Health Survey, which is a well-established and validated instrument [18, 19]. Because improvements to dietary quality (e.g., increased consumption of vegetables) have been found to be positively associated with self-reported health [20–22], we hypothesized that women who received the healthier revised WIC food package would have better self-reported health. Second, we assessed body mass index (BMI), which was calculated using a woman’s self-reported height and weight. Prior research has demonstrated the benefits of the revised WIC food package on reduced gestational weight gain [14], as well as breastfeeding [23], which is associated with reduced postpartum weight retention. Furthermore, excessive weight gain during pregnancy has been found to be predictive of long-term obesity in women [24, 25]. Therefore, we hypothesized that receiving the healthier WIC food package could manifest in reductions in women’s post-partum BMI.

For children, we evaluated several indicators of physical and mental health. First, we evaluated anemia, which was defined based on an affirmative response to the question, “During the past 12 months, has [child] had anemia?” Anemia is a critical health outcome in early childhood and has been found to impact children’s cognitive outcomes [26, 27] and long-term growth [28], and prior studies have found that receiving WIC benefits was associated with increased iron intake for children [29, 30]. Second, we evaluated change in parent-reported health similar to the question for adults described above. Finally, results from a systematic review suggest that improvements to overall dietary quality led to improvements in mental health for children [31], while another study found that the 2009 WIC revision improved child development outcomes among recipient children [16]. Therefore, we additionally evaluated changes to children’s mental health, which was measured for children aged 2–3 years with the Mental Health Indicator (MHI) score. The MHI is a validated tool adapted from the Child Behavior Checklist and includes questions about whether a child had trouble sleeping, was unhappy/depressed, or was nervous/high-strung during the previous two months [32]. Although there was slight variation in the questions asked of girls and boys, the MHI questions were intended to measure the same construct, so scores across sex were pooled into one variable, as has been done in previous studies [33, 34]. Higher scores on the MHI score (range 0–8) indicate increased risk for mental health problems.

### Covariates

All models adjusted for covariates that might confound the relationship between receipt of the revised WIC food package and the outcomes, including age, parent marital status, family size, parental education, race/ethnicity (to capture experiences of structural or interpersonal racism), and inflation-adjusted family income. For race/ethnicity, we used the categories of White, Black, Hispanic, and other (including Asian American, American Indian, and those who did not further self-identify a specific race or ethnicity). The latter category is a heterogeneous group for which effect estimates may be difficult to interpret, although we were not able to create more granular categories due to small cell sizes and unstable estimates. Fixed effects for state were included to account for time-invariant characteristics of states that may have confounded the relationship between the state’s timing of policy implementation and the outcomes of interest, and fixed effects for year accounted for secular trends.

### Primary analysis

We first calculated descriptive statistics stratified by women’s or children’s receipt of WIC and whether the interview was conducted before or after the revised WIC food package was implemented. Then, we estimated the effect of the revised WIC food package on women’s and children’s health outcomes using difference-in-differences (DID) analysis. DID is a quasi-experimental approach that compares trends in a given outcome in a “treatment” group before and after the implementation of a policy, while “differencing out” the secular trends in the outcome in a “control” group of individuals unaffected by the policy [35]. We leveraged the fact that the WIC food package revisions were unlikely to be associated with the characteristics of individuals in our sample. In brief, DID analysis involves a multivariable linear regression model in which the primary predictor is an interaction term between a binary variable for WIC receipt and an indicator for whether the interview was conducted after the revision. DID estimation requires several assumptions to produce valid estimates. Importantly the baseline characteristics of the treatment and control groups do not have to be the same, but rather the trends in outcomes for the treatment and control groups during the pre-revision period must be similar (i.e., the “parallel trends” assumption). Further details, including the equation, are provided in the Supplementary file 1.

### Secondary analyses

We conducted several subgroup analyses to evaluate differential responses to the revised WIC food package. For both women’s and children’s outcomes, we evaluated heterogeneity in estimates by race/ethnicity (White, Black, Hispanic, other), parental education (high school or less versus some college or more), and women’s/mother’s age (under 35 versus 35 and older).

Additional sensitivity analyses were conducted to test the robustness of the results. First, since self-report of safety net benefit receipt can be unreliable [36, 37], we conducted a sensitivity analysis in which the primary exposure was based on WIC eligibility rather than actual receipt, akin to an intent-to-treat design. We imputed eligibility for WIC on state, year, household size, self-reported income, presence of children under one in the household (for women’s eligibility), and age under five (for children’s eligibility). Because income and household size were self-reported in NHIS and might not correspond to the information provided to WIC to determine eligibility, this approach may result in measurement error and therefore was not considered the primary analysis. Second, we included fixed effects for the interview month to provide a more granular adjustment for secular trends. Third, we evaluated whether results were sensitive to group-specific linear time trends, by including an interaction term for WIC receipt and a continuous variable for time. In effect, including group-specific trends in the main model allows outcome trajectories to differentially change over time, allowing for a relaxation of the parallel trends assumption of DID analysis. Finally, it is possible that women may still benefit from their children’s participation in WIC even if they do not receive benefits themselves, since food might be distributed throughout the household. Thus, for the women’s analysis, we included a sensitivity analysis where we redefined the sample to those with children under the age of five.

## Results

### Sample characteristics

The final sample included 81,771 women and 27,780 children (Table 1). Overall, a higher proportion of WIC recipients reported completing high school or less education (63.2% of women, 61.9% of children’s parents) compared with non-recipients (42.5% of women, 45.9% of children’s parents). WIC recipients were also more likely to be Hispanic (30.1% of women, 40.3% of children) compared with non-recipients (21.7% of women, 26.6% of children). Indicators of women’s health were similar for WIC recipients and non-recipients, while children’s MHI scores were higher among recipients compared with non-recipients in both periods before and after the revision. Importantly, DID does not require that characteristics of the treatment and control group be similar, but rather that trends (i.e., slopes) in outcomes be parallel during the pre-revision period, as described below.

**Table 1:** Sociodemographic and health characteristics, by WIC receipt and interview date

	WIC, % or mean (SD)		No WIC, % or mean (SD)	
**Women**	**Before revision** **(n = 2948)**	**After revision** **(n = 2750)**	**Before revision** **(n = 38,877)**	**After revision** **(n = 37,196)**
Age (years)	27.2 (6.0)	27.9 (6.0)	31.6 (7.8)	31.3 (7.7)
Marital status	43.9	40.0	35.0	30.9
Size of family	3.8 (1.6)	3.9 (1.6)	2.8 (1.6)	2.8 (1.6)
Educational attainment				
Less than high school	34.7	26.2	19.1	15.1
High school	33.3	32.0	27.5	23.4
Some college	28.0	35.1	35.4	38.3
College or more	4.0	6.7	17.9	23.3
Race/Ethnicity				
White	33.9	36.9	49.3	47.8
Black	26.0	24.0	21.1	19.5
Hispanic	30.4	29.7	21.1	22.3
Other	9.7	9.3	8.5	10.4
Family income	25,935 (17,267)	25,437 (17,788)	35,181 (20,397)	32,721 (20,618)
Body mass index	27.8 (6.6)	28.7 (6.8)	26.9 (6.6)	27.7 (7.0)
Self-reported health	0.9 (0.3)	0.9 (0.3)	0.9 (0.3)	0.9 (0.3)
**Children**	**Before revision** **(n = 4322)**	**After revision** **(n = 5578)**	**Before revision** **(n = 8285)**	**After revision** **(n = 9595)**
Age (years)	1.7 (1.3)	1.8 (1.3)	2.1 (1.4)	2.1 (1.4)
Mom’s marital status	57.3	51.0	65.5	60.0
Size of family	3.9 (1.4)	4.0 (1.4)	3.8 (1.3)	3.8 (1.3)
Parent educational attainment				
Less than high school	35.7	29.1	23.6	15.1
High school	31.3	31.9	29.3	27.1
Some college	27.3	31.5	32.7	37.3
College or more	5.7	7.4	14.4	20.6
Race/Ethnicity				
White	26.7	28.7	42.5	44.9
Black	20.6	20.2	18.1	17.1
Hispanic	42.1	38.8	28.4	25.0
Other	10.6	12.3	11.1	13.0
Family income	29,398 (17,465)	27,799 (17,489)	40,471 (20,192)	39,426 (20,669)
Anemia	0.0 (0.2)	0.0 (0.2)	0.0 (0.1)	0.0 (0.1)
Parent-reported health	1.0 (0.2)	1.0 (0.1)	1.0 (0.1)	1.0 (0.1)
Mental Health Indicator	1.6 (1.7)	1.4 (2.6)	1.4 (1.6)	1.2 (2.1)

The study sample was drawn from the National Health Interview Survey for years 1998–2017. Abbreviations: SD, standard deviation; WIC, Special Supplemental Nutrition Program for Women, Infants, and Children

### DID model assumptions

In qualitative assessments of the parallel trends assumption, women’s health outcomes demonstrated roughly parallel slopes for recipients and non-recipients in the period prior to the revision (Supplemental Fig. 2). Quantitative evaluations of parallel trends further confirmed that this assumption was reasonably fulfilled (Supplemental Table 1). We next analyzed whether there were differential compositional changes after the policy revision among recipients compared with non-recipients (Supplemental Table 2). We observed differences in key demographic and socioeconomic factors, which were not consistent in magnitude or direction across the two samples. While we adjusted for these factors in our analysis, it is possible that there are also differences in unobserved characteristics that may result in residual confounding, a limitation of all DID analyses.

### Association of revised WIC food package with women’s and children’ health

For both women and children, we were unable to rule out the null hypothesis that there was no association between receipt of the revised WIC food package and the health outcomes of interest (Table 2). Results from all sensitivity analyses were consistent with findings from the primary analysis.

**Table 2:** Association of the revised WIC food package with women’s and children’s health

	Primary analysis	WIC eligible	Month-specific fixed effects	Group-specific trends	Household kids under 5
**Women’s health outcomes**					
Body mass index	-0.14 [-0.57, 0.30]	-0.15 [-0.57, 0.27]	-0.13 [-0.56, 0.30]	0.22 [-0.69, 1.12]	-0.14 [-0.57, 0.30]
Self-reported health	-0.00 [-0.01, 0.02]	-0.01 [-0.03, 0.01]	0.00 [-0.01, 0.02]	-0.02 [-0.04, 0.01]	0.00 [-0.01, 0.02]
**Children’s health outcomes**					
Anemia	0.00 [-0.01, 0.00]	0.00 [-0.00, 0.01]	0.00 [-0.01, 0.00]	-0.01 [-0.02, 0.01]	--
Parent-reported health	0.00 [-0.00, 0.00]	0.00 [-0.01, 0.01]	0.00 [-0.00, 0.01]	0.00 [-0.02, 0.01]	--
Mental Health Indicator	0.05 [-0.23, 0.14]	-0.09 [-0.28, 0.10]	-0.04 [-0.24, 0.15]	-0.02 [-0.40, 0.37]	--

Note: The study sample was drawn from the National Health Interview Survey for years 1998–2017. Difference-in-differences analysis was conducted adjusting for age, parent marital status, family size, parental education, race/ethnicity, and family income, and additionally included fixed effects for state and year. Values above represent the coefficients and 95% confidence interval on the interaction term between WIC receipt and whether the interview date occurred after the implementation of the WIC food package revision, thereby capturing the effect of the revised WIC food package on each outcome. Abbreviations: WIC, Special Supplemental Nutrition Program for Women, Infants, and Children

Results from the stratified analysis additionally revealed that there were no differences in effect estimates by race/ethnicity, educational attainment, or age (Supplemental Table 3).

## Discussion

Our study provides evidence for the effects of the revised WIC food package on several indicators of health for women and children. By using a quasi-experimental approach and a national repeated cross-sectional sample, we extend current knowledge about the potential effects of the WIC food package revision on downstream women’s and children’s outcomes. Across all indicators of health that we evaluated, coefficients were small, and we were unable to rule out the null hypothesis that there were no effects of the revised WIC food package.

Our results on downstream health are inconsistent with prior research that has examined the impacts of the WIC revision on nutritional outcomes. For instance, prior studies have found that the revision led to improvements in proximal outcomes including dietary quality and access to healthy foods [6, 9]. Additionally, a smaller number of studies have looked at perinatal health in sub-national samples [14–16]. As a specific example, one previous study found positive improvements in gestational weight gain following the revision [14]. In contrast, we failed to detect a significant association between the revised WIC food package and women’s BMI. There are several potential explanations. First, women are only eligible for WIC during pregnancy and up to six months postpartum (for non-breastfeeding women) or one year postpartum (for breastfeeding women). Given the short duration of eligibility for women and the modest scope of the WIC revision, it is possible that improvements that have previously been found for gestational weight gain do not translate into longer-term changes to BMI in the absence of longer-term nutrition support. This presents a potential area in which the WIC program can continue to expand and improve its services. For instance, a recent pilot study evaluating the effects of a cost-neutral, integrated WIC and obstetrical service model found integrated care to be a promising approach for limiting postpartum weight retention [38]. Furthermore, while the revised WIC food package has been found to improve dietary quality and nutrient intake for women during pregnancy [6], the duration of WIC services for women may need to be longer in order to influence BMI beyond improving nutrition during pregnancy. It is also possible that there is a high level of measurement error in BMI during the postpartum period because of rapid changes to weight during this window, which may contribute to null results.

Prior research on the effects of the WIC program (more generally, not just the revision) on longer-term health outcomes has been somewhat inconsistent. For instance, evidence on effects of overall WIC participation on children’s general health status and anemia has been mixed. For instance, while some studies have found positive associations between WIC participation and self-reported assessments of children’s general health [39, 40], others have found no associations [41]. For anemia, while a descriptive study using data from the Pediatric Nutrition Surveillance System found a reduction in the prevalence of anemia among low-income children [42], more recent findings from a convenience sample of children recruited from WIC clinics in California found WIC participation was negatively associated with both anemia and iron deficiency [43]. That said, much of this prior research was either descriptive, or compared outcomes between WIC recipients (at high nutritional risk) with non-recipients and was therefore limited by bias due to confounding. Additionally, practically speaking, inconsistencies in existing research may reflect differences in sample populations, study design, and measurement of variables. In our current study, we overcame the limitations of these previous investigations by using quasi-experimental techniques, and we were unable to reject the null that there was no effect of the 2009 WIC revision on these health outcomes. Only one other study exists that evaluates the effect of the 2009 revision on childhood anemia, using quasi-experimental methods and data from the National Health and Nutrition Examination Survey [44], in which investigators found reductions in the probability of anemia in children participating in WIC. There are several reasons why our results may differ. First, data on anemia was collected differently between the two studies. While data on anemia are self-reported in the NHIS, NHANES data included biologic samples, enabling greater precision in definition of disease. Second, while the former study defined their sample as children aged 2–5, our study additionally included infants. Altogether, considering mixed evidence of the effect of WIC participation (generally) on childhood anemia, our findings reaffirm the importance of conducting additional research to replicate and further elucidate the effects of the 2009 revisions.

Strengths of this study include the use of a quasi-experimental study design. Additionally, we used a national repeated cross-sectional dataset, while most prior studies of the WIC revision have been conducted in more limited geographies. Our study also has several limitations. First, self-reported outcomes and covariates, including WIC benefit receipt, are subject to standard reporting biases and may result in measurement error [37]. Misclassification of our exposure variable, if non-differential, typically tends to bias estimates toward the null, and may have contributed to our overall null findings. Nevertheless, imputation of safety net benefits is something that is commonly conducted [45–47], since merging in administrative data on program participation is often logistically infeasible. Additionally, because all outcome variables were self-reported, it is possible that heterogeneous effects of different outcome types could have been masked. For instance, because the NHIS did not collect data on different etiologic manifestations of anemia (i.e., iron-deficient, thalassemia, or sickle cell), this variable was evaluated generally. It is also possible that caregivers are not aware of whether their child has anemia, so future studies should examine this outcome using objectively measured serum tests. Furthermore, while we did not observe a statistically significant effect of the WIC revision on self-reported health, it is possible that this outcome was not specific enough to adequately gauge the impact of the modest changes to WIC, or that it covered too long of a time (12 months). Although dietary quality has been found to be associated with improvements in individual self-rated health, it is very possible that a lack of consistent participation in WIC and changes to perceptions of health during pregnancy and post-partum masked any meaningfully observable effects of the modest revisions. Moreover, use of a dichotomous measure of past-year WIC receipt does not consider history of prior WIC participation (or possible sporadic WIC participation throughout the current year). Because changes to outcomes may result from cumulative participation, future studies should evaluate whether duration of WIC participation impacted outcomes differentially before and after the revision. Additionally, DID analysis rests of the assumption that there are no other contemporaneous policy or intervention that would have differentially affected recipients and non-recipients. Notably, the WIC revision occurred during the Great Recession, and prior work has shown that lower-income individuals were more adversely affected; this possible confounding event might also drive results toward the null, although prior studies similar to ours nevertheless detected a positive effect of the WIC revision.

In summary, we found no longer-term effects of the 2009 revised WIC food package on indicators of physical and mental health for women or children. This suggests that while the modest revisions to the WIC food package have had a proximal impact on nutrition, diet, and perinatal outcomes, the changes may be too modest or short-lived to result in meaningful changes in downstream health. As policymakers consider expansions and revisions of WIC, particularly in the wake of the COVID-19 pandemic, it is critical to assess the impact of WIC revisions to inform these efforts. Specifically, as a part of the American Rescue Plan Act of 2021, the cash voucher benefits for fruit and vegetable purchases were temporarily increased to $35 per child and adult per month (from $11 for women and $9 for children) [48]. Additionally, in response to the pandemic, the USDA granted waivers to state agencies to enable continued access. As a result, nearly all (99%) of WIC agencies conducted remote certification appointments (compared to 12% prior to the pandemic) [49]. The impacts of these amendments to the WIC program, which were substantially larger than the 2009 revision, need to be evaluated. Efforts to modify the program will be critical to fulfill its mission of achieving health equity for low-income and vulnerable families.

## Key messages

### Practice

Community organizations, researchers, and clinicians should advocate for continued improvement and expansion of WIC so that nutritional interventions keep pace with the dynamic needs of low-income families.

### Policy

While this study did not find associations between the 2009 revision on longer-term indicators of physical and mental health, prior studies which have identified impacts of the revision on more proximal outcomes suggest that more expansive modifications to the program are needed to influence longer-term health outcomes.

### Research

Following the more substantial expansions to the WIC program during the COVID-19 pandemic, future studies are needed to understand whether these alterations led to long-term improvements in population health.

## Electronic supplementary material

Below is the link to the electronic supplementary material.

Supplementary Material 1

## Data Availability

Data described in the manuscript, codebook, and analytic code will not be made available because analysis was conducted using restricted geographic data, which could lead to the identification of participants. Interested investigators can apply for these data at https://www.cdc.gov/rdc.

## Abbreviations

BMI: Body mass index.
DID: Difference-in-differences.
MHI: Mental Health Indicator.
NHIS: National Health Interview Survey
WIC: Special Supplemental Nutrition Program for Women, Infants, and Children.
