# Supplementary material for: Effects of the revised WIC food package on women’s and children’s health: a quasi-experimental study
Source: BMC Pregnancy Childbirth. 2022 Nov 2;22:806. doi: 10.1186/s12884-022-05116-w (PMC9628263; doi:10.1186/s12884-022-05116-w)
Supplement: Supplementary file 1 — Supplementary Material 1 [file 12884_2022_5116_MOESM1_ESM.pdf]

SUPPLEMENTAL MATERIALS

Difference-in-differences analysis

The effect of the revised food package on women's and children's health outcomes was estimated using difference-in-differences (DID) analysis. The equation for this model for each outcome Y was specified as follows, for each individual *i* (woman or child) in time *t* in state *s*:

$$Y_{its} = \beta_0 + \beta_1 WIC_{it} \times Post_t + \beta_2 WIC_{it} + \beta_3 Post_t + \beta_4 Covar_{it} + \beta_5 State_s + \beta_6 Year_t + \varepsilon_{its}$$

β_1 is the coefficient of interest in the DID model and represents the effect of the revised WIC food package on the outcome Y. $Covar$ represents a vector of women's or children's covariates, $State$ represents state fixed effects, and $Year$ represents year fixed effects.

We used linear models for both continuous and binary outcomes. Linear models are preferred for DID analyses due to differences in the interpretation of interaction terms in non-linear models (1,2). For binary outcomes, this means that the regression represents a linear probability model, and the DID effect is therefore interpreted as a percentage-point change in risk.

Several key assumptions underly DID analyses. First, DID estimation relies on the assumption that the outcome trends in the period following the revision would have been the same for WIC recipients and non-recipients had the food package not been revised. Although this counterfactual condition cannot be observed, several analyses were conducted to evaluate the validity of this assumption. We first qualitatively inspected whether the trends in outcomes among recipients and non-recipients were parallel during the period prior to the revision (Supplemental Figure 2). We additionally tested the parallel trends assumption quantitatively, by restricting the data to the period before the WIC food package revision was implemented and

including an interaction term between a binary variable for WIC receipt and a continuous variable for time (Supplemental Table 1). A null effect in this analysis would reassure us of no statistically significant difference in the pre-revision parallel trends.

Second, DID estimation assumes that any observed effects are not due to differential changes in the underlying composition of the treatment versus the control groups. To test this assumption, we evaluated whether characteristics in the women and children samples differed between the pre- and post-revision periods. This was done by modeling each covariate as the dependent variable in separate regression models, in which the primary predictor is the interaction term between an indicator for WIC receipt and an indicator for whether the interview was conducted after the revision was implemented (Supplemental Table 2).

SUPPLEMENTAL REFERENCES

1. Athey S, Imbens GW. Identification and Inference in Nonlinear Difference-in-Differences Models. Econometrica. 2006;74(2):431–97.
2. Karaca-Mandic P, Norton EC, Dowd B. Interaction terms in nonlinear models. Health Serv Res. 2012 Feb;47(1 Pt 1):255–74.

Supplemental Figure 1. Sample flowchart

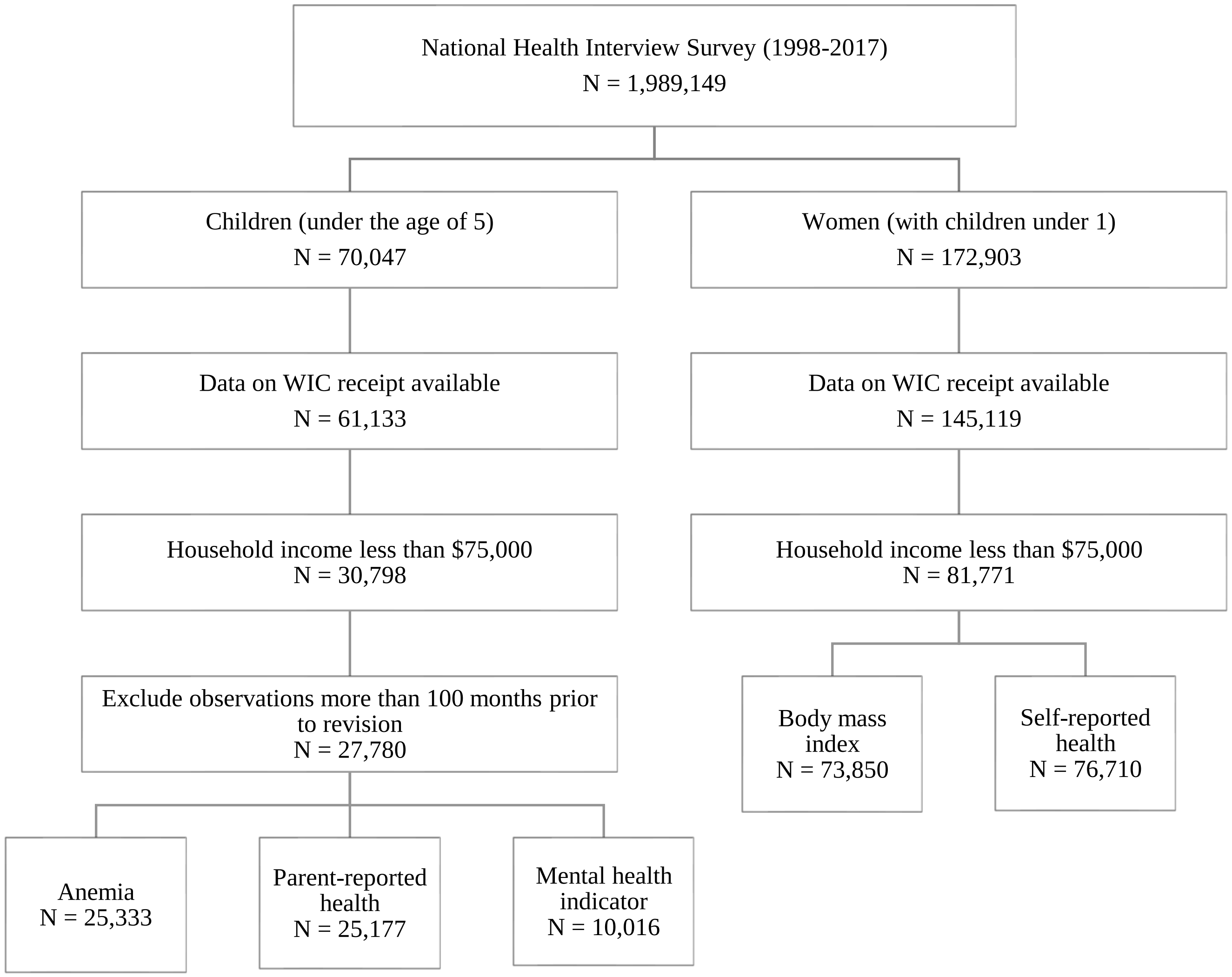

Note: Final sample sizes for available outcomes additionally account for covariates available in final models.
Abbreviations: WIC, Special Supplemental Nutrition Program for Women, Infants and Children

Supplemental Figure 2. Graphical evaluation of the parallel trends assumption for women's and children's health outcomes

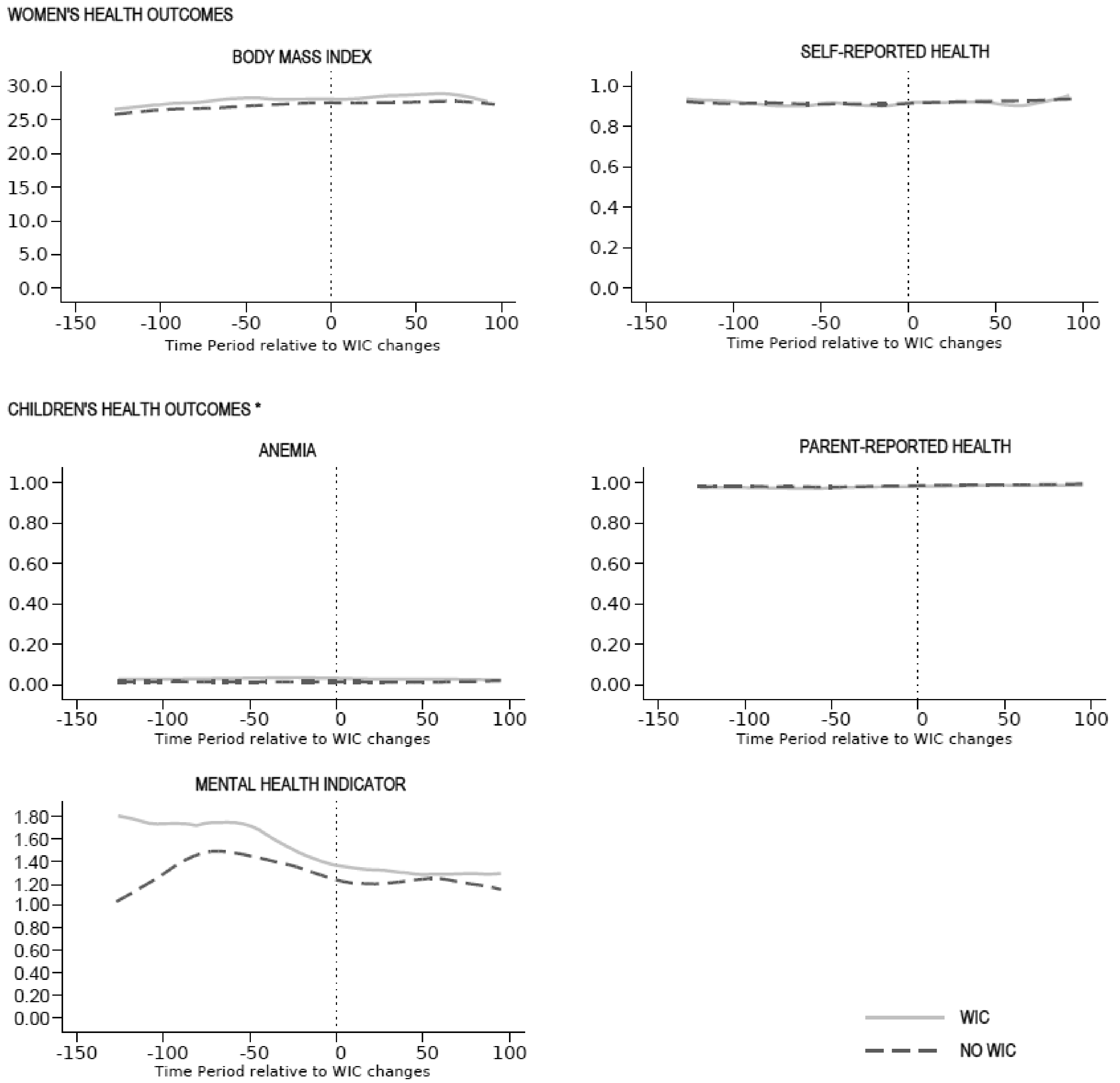

Note: The study sample was drawn from the National Health Interview Survey for years 1998-2017. The implementation of the revised WIC food package was staggered across states during 2009, ranging from January to November. The dotted vertical line on each graph indicates when the revised WIC food package was revised for each individual's state of residence. Abbreviations: WIC, Special Supplemental Nutrition Program for Women, Infants, and Children

* For children's health outcomes, we removed observations prior to 100 months before the WIC revision because trends in earlier periods were not parallel, as shown in this Figure.

Supplemental Table 1. Quantitative assessment of parallel trends assumption for women and children

	β	95% CI
**Women's health outcomes**		
Body mass index	0.00	[-0.01, 0.00]
Self-reported health	0.00	[-0.00, 0.00]
**Children's health outcomes**		
Anemia	0.00	[-0.00, 0.00]
Parent-reported health	0.00	[-0.00, 0.00]
Mental Health Indicator	0.00	[-0.01, 0.00]

Note: The study sample was drawn from the National Health Interview Survey for years 1998-2017. We regressed each outcome on an indicator for WIC receipt, a variable representing the difference between interview date and date of implementation (computed based on state and year for each individual), and an interaction between the two. The above estimates represent the coefficient on the interaction term. A non-statistically significant coefficient provides evidence of similarity in the trends between recipients and non-recipients before the revision was implemented. Abbreviations: WIC, Special Supplemental Nutrition Program for Women, Infants, and Children

Supplemental Table 2. Association of the revised WIC food package implementation with participants' characteristics

	β	95% CI
**Women's characteristics**		
Age	1.07***	[0.66, 1.48]
Marital status	0.00	[-0.02, 0.03]
Size of family	0.06	[-0.02, 0.15]
Education: High school	0.03**	[0.00, 0.05]
Education: Some college	0.04***	[0.02, 0.07]
Education: College or more	-0.03**	[-0.05, -0.00]
Race: Black	0.00	[-0.03, 0.02]
Race: Hispanic	-0.02	[-0.04, 0.00]
Race: Other	-0.02***	[-0.04, -0.01]
Inflation-adjusted family income	1963.31***	[869.09, 3057.53]
**Children's characteristics**		
Age	-0.38***	[-0.43, -0.33]
Marital status	-0.08***	[-0.10, -0.06]
Size of family	0.14***	[0.09, 0.19]
Education: High school	0.02**	[0.00, 0.04]
Education: Some college	-0.05***	[-0.07, -0.04]
Education: College or more	-0.09***	[-0.10, -0.07]
Race: Black	0.03***	[0.01, 0.04]
Race: Hispanic	0.14***	[0.12, 0.15]
Race: Other	0	[-0.02, 0.01]
Inflation-adjusted family income	-11072.20***	[-11787.26, -10357.13]

*** p<0.01, ** p<0.05

Note: The study sample was drawn from the National Health Interview Survey for years 1998-2017. The above coefficients represent the interaction between a binary variable for being interviewed in the time period after the WIC revision was implemented and an indicator for whether or not the participant was a WIC recipient. These models examine whether there are differences in demographic characteristics between recipient/non-recipients and eligible/non-eligible individuals before and after the implementation of the food package revision. Null results indicate that there are no differences in sample composition for a given covariate. Abbreviations: WIC, Special Supplemental Nutrition Program for Women, Infants, and Children

Supplemental Table 3. Associations of the revised WIC food package with women's and children's health outcomes, by race/ethnicity, parental education, and maternal age

	Results stratified by race/ethnicity				Results stratified by parental education[a]		Results stratified by mother's age[b]	
	**White**	**Black**	**Hispanic**	**Other**	**HS or less**	**College or more**	**Under 35**	**35 and older**
**Women's health outcomes**								
Body mass index	-0.51 [-1.16, 0.15]	-0.60 [-1.41, 0.21]	0.52 [-0.08, 1.13]	0.64 [-0.34, 1.63]	-0.01 [-0.55, 0.54]	-0.41 [-1.00, 0.18]	-0.19 [-0.64, 0.25]	0.71 [-0.50, 1.92]
Self-reported health	0.02 [-0.01, 0.05]	-0.01 [-0.05, 0.02]	0.00 [-0.02, 0.03]	-0.02 [-0.07, 0.03]	-0.01 [-0.03, 0.01]	0.02 [-0.01, 0.04]	0.00 [-0.01, 0.02]	0.00 [-0.04, 0.03]
**Children's health outcomes**								
Anemia	-0.01 [-0.02, 0.01]	0.00 [-0.02, 0.02]	0.00 [-0.01, 0.00]	-0.01 [-0.02, 0.01]	0.00 [-0.01, 0.01]	-0.01 [-0.02, 0.00]	-0.01 [-0.01, 0.00]	0.01 [-0.01, 0.03]
Parent-reported health	0.01 [-0.00, 0.02]	-0.01 [-0.03, 0.01]	0.00 [-0.01, 0.02]	0.00 [-0.01, 0.02]	0.00 [-0.01, 0.01]	0.00 [-0.01, 0.02]	0.00 [-0.01, 0.01]	0.01 [-0.01, 0.03]
Mental Health Indictor	0.21 [-0.30, 0.72]	-0.01 [-0.34, 0.31]	-0.11 [-0.29, 0.06]	-0.38 [-0.87, 0.12]	0.01 [-0.27, 0.28]	-0.01 [-0.26, 0.23]	-0.05 [-0.24, 0.14]	-0.01 [-0.43, 0.40]

Note: The study sample was drawn from the National Health Interview Survey for years 1998-2017. Difference-in-differences analysis was conducted adjusting for age, parent marital status, family size, parental education, race/ethnicity, and family income, and additionally included fixed effects for state and year. Values above represent the coefficients on the interaction term between WIC receipt and whether the interview date occurred after the implementation of the WIC food package revision. These coefficients capture the effect of the revised WIC food package on each of the health indicators of interest. Abbreviations: WIC, Special Supplemental Nutrition Program for Women, Infants, and Children; HS, high school

[a] Refers to parent's educational attainment for children's sample
[b] Refers to own age for women's sample
